# Identification of Aberrantly Expressed miRNAs in Gastric Cancer

**DOI:** 10.1155/2014/473817

**Published:** 2014-06-01

**Authors:** Dan Liu, Xiaowei Hu, Hongfeng Zhou, Guangyue Shi, Jin Wu

**Affiliations:** The Seventh Department of Internal Medicine, The Affiliated Tumor Hospital of Harbin Medical University, Harbin, Heilongjiang 150081, China

## Abstract

The noncoding components of the genome, including miRNA, can contribute to pathogenesis of gastric cancer. Their expression has been profiled in many human cancers, but there are a few published studies in gastric cancer. It is necessary to identify novel aberrantly expressed miRNAs in gastric cancer. In this study, the expression profile of 1891 miRNAs was analyzed using a miRCURY array LNA miRNA chip from three gastric cancer tissues and three normal tissues. The expression levels of 4 miRNAs were compared by real-time PCR between cancerous and normal tissues. We found that 31 miRNAs are upregulated in gastric cancer (*P* < 0.05) and 10 miRNAs have never been reported by other studies; 30 miRNA are downregulated (*P* < 0.05) in gastric cancer tissues. Gene ontology analysis revealed that those dysregulated miRNAs mainly take part in regulating cell proliferation. The levels of has-miR-105, -213∗, -514b, and -548n were tested by real-time PCR and have high levels in cancerous tissues. Here, we report a miRNA profile of gastric cancer and provide new perspective to understand this malignant disease. This novel information suggests the potential roles of these miRNAs in the diagnosis, prognosis biomarkers, or therapy targets of gastric cancer.

## 1. Introduction

Gastric cancer is one of the most frequent cancers and is the second leading cause of cancer mortality worldwide [1]. Nearly half of gastric cancers occur in China, most of which are diagnosed when the disease has progressed to late stages because of the nonspecific symptoms present at early stages [2]. As a result, the overall 5-year survival rate of gastric cancer was approximately 20% [3]. Tumor markers have the potential to improve the situation by screening high risk group at early stage. Unfortunately, the tumor markers, such as carcinoembryonic antigen (CEA), carbohydrate antigen 19-9 (CA19-9), CA12-5, and CA72-4, have limitation performance in detecting gastric cancer because of low sensitivity and specificity [4]. Thus, there is a need to discover some novel diagnostic biomarkers to allow early detection of this malignancy. It is known that several environmental factors, including diet high in salted and nitrated food, tobacco use, alcoholic consumptions, and, especially,* Helicobacter pylori* (HP) infection [5]. However, the molecular pathogenesis of gastric cancer still remains to be explored. Mechanisms for tumorigenesis and progression of gastric cancer have not yet been discovered and specific therapeutic targets have not been identified [6].

Micro-RNAs (miRNAs) are small noncoding regulatory RNAs of about 19–22 nucleotides, which function to bind the 3′ untranslated region of their target mRNAs, resulting in translational inhibition or mRNA degradation [7]. While the biological roles of miRNA are under intense investigation, they are considered to control a variety of tumor cell functions including cell proliferation, migration, invasion, and differentiation [8]. A growing number of evidences suggest the correlation altered miRNA expression and cancers. The aberrant expression of miRNAs referred to several important processes during carcinogenesis. Let-7 is one of the earliest identified miRNAs, is significantly reduced in a large number of malignancies, and can attenuate the development of lung cancer [9]. High-throughput techniques, such as gene chip, have identified thousands of upregulations such as miR-21, miR-17, and miR-92a, whereas others tended to downregulation, such as miR205 and miR-145, in cancerous tissues [10]. Several studies have investigated the aberrantly expressed miRNAs in gastric cancer. miR-199a-3p is significantly higher in serum of gastric cancer patients and may be used as diagnostic biomarker [4]. MiR-34a was downregulated in gastric cancer cell lines [11, 12]. MiR-429 suppresses tumor cells proliferation and may serve as tumor suppressor during tumorigenesis of gastric cancer [13]. MiR-19a/b regulates multidrug resistance in gastric cancer by targeting PTEN [14]. Li et al. had identified abnormal expressed miRNA profiles with 40 upregulated miRNAs and 36 downregulated miRNAs in intestinal-type gastric caners by miRNA array [6]. It has been estimated that there are about 1,000 miRNAs in human, but previous studies have only screened miRNA expression in gastric cancer patients from less than 500 miRNAs. It is necessary to screen gastric cancer with larger collections of miRNAs by gene chip.

To explore more related miRNAs, we have used 6th generation of miRNA array that contains more than 1891 probes, which include nearly all human miRNAs and identified new aberrant expression miRNAs between gastric cancer and normal tissues. We identified 61 new obviously changed expression miRNAs in gastric cancers. Our data offer new clues to study the miRNA profiles that refer to molecular mechanism of gastric cancer carcinogenesis and provide novel biomarkers repertoire of this malignant disease.

## 2. Material and Methods

The tissue samples in this study were derived from patients undergoing a surgical procedure to remove a portion of gastric cancer at the Affiliated Tumor Hospital of Harbin Medical University. The collection of samples conformed to the policies of China and practices of the facility's Institutional Review Board. Upon removal of the surgical specimen, research personnel immediately transported the tissue to the surgical pathology laboratory. Pathology faculty performed a gross analysis of the specimen and selected cancerous appearing gastric tissue and normal appearing gastric cancer for research. Each sample was placed in a cryovial and preserved in −80°C refrigerator until analysis. Subsequently the surgical specimens confirmed the histopathology of the samples taken for research.

### 2.1. Total RNA Isolation and Quality Analysis

Frozen tissues were first pulverized in a stainless steel mortar and pestle. Total RNA was isolated using TRIzol (Invitrogen, Carslbad, CA) and miRNeasy mini kit (QIAGEN) according to manufacturer's instructions. RNA quality and quantity were measured by using nanodrop spectrophotometer (ND-1000, Nanodrop Technologies) and RNA integrity was determined by gel electrophoresis.

### 2.2. miRNA Precursor Expression Profiling

After RNA isolation from the samples, the miRCURY Hy3/Hy5 power labeling kit (Exiqon, Vedbaek, Denmark) was used according to the manufacturer's guideline for miRNA labelling. One microgram of each sample was 3′-end-labeled with Hy3 fluorescent label, using T4 RNA ligase by the following procedure: RNA in 2.0 *μ*L of water was combined with 1.0 *μ*L of CIP buffer and CIP (Exiqon). The mixture was incubated for 30 min at 37°C and was terminated by incubation for 5 min at 95°C. Then 3.0 *μ*L of labeling buffer, 1.5 *μ*L of fluorescent label (Hy3), 2.0 *μ*L of DMSO, and 2.0 *μ*L of labeling enzyme were added into the mixture. The labeling reaction was incubated for 1 h at 16°C and terminated by incubation for 15 min at 65°C. After stopping the labeling procedure, the Hy3-labeled samples were hybridized on the miRCURY LNA array (v.16.0) (Exiqon) according to array manual. The total 25 *μ*L mixture from Hy3-labeled samples with 25 *μ*L hybridization buffer was first denatured for 2 min at 95°C, incubated on ice for 2 min, and then hybridized to the microarray for 16–20 h at 56°C in a 12-Bay Hybridization Systems (Hybridization System, Nimblegen Systems, Inc., Madison, WI, USA), which provides an active mixing action and constant incubation temperature to improve hybridization uniformity and enhance signal. Following hybridization, the slides were achieved, washed several times using Wash buffer kit (Exiqon), and finally dried by centrifugation for 5 min at 400 rpm. Then the slides were scanned using the Axon GenePix 4000B microarray scanner (Axon Instruments, Foster City, CA).

### 2.3. miRNA Quantification by Real-Time RT-PCR (qRT-PCR)

SYBR Green RT-qPCR assay was used for miRNA quantification. In brief, one microgram of extracted RNA was reverse-transcribed per the manufacturer's instructions. qRT-PCR was carried out in thin-wall PCR plates (Applied Biosystems, Foster City, CA, USA). Each reaction mixture contained 12.5 *μ*L of SYBR Premix Ex Taq (2×), 0.5 lL of reference dye (ROX) II (50×) (Takara, Otsu, Japan), 0.5 *μ*L of forward primer (10 *μ*M), 0.5 *μ*L of reverse primer (10 *μ*M), 10.7 *μ*L of distilled water, and 0.3 *μ*L of cDNA template. PCR was carried out by following the standard PCR program suggested by the manufacturer's protocol using Mx3000P (Stratagene, La Jolla, CA, USA).

### 2.4. Data Analysis

Scanned images were then imported into GenePix Pro 6.0 software (Axon) for grid alignment and data extraction. Replicated miRNAs were averaged and miRNAs with intensities >50 in all samples were chosen for calculating normalization factor. Expressed data were normalized using the median normalization. After normalization, differentially expressed miRNAs were identified through volcano plot filtering. Hierarchical clustering was performed using MEV software (v4.6, TIGR). GoStat was used to determine all genes with statistically overrepresented gene ontology (GO) annotation [15].

## 3. Results

### 3.1. miRNA Expression Profiles in Gastric Cancer

We used miRCURY array LNA miRNA chip, which contains more than 1891 capture probes, to evaluate miRNA expression profiles between cancerous tissues and normal tissues. When setting average change >2-fold and *P* value <0.05 as a cut-off level, 31 miRNAs are upregulated (Table 1) and 30 miRNAs are downregulated in gastric cancers (Table 2). Comparing with previous studies of miRNAs, we found that 21 among 31 genes have been reported in previous publications, for example, hsa-miR-105, hsa-miR-187, hsa-miR-214*, hsa-miR-656, and hsa-miR-487a. New upregulated genes include named genes such as hsa-miR-4309, hsa-miR-4307, hsa-miR-4278, and hsa-miRPlus-C1114. Twenty-six among 30 downregulated genes have been reported before such as hsa-miR-31, hsa-miR-1275, hsa-miR-526b, hsa-miR-2114, and hsa-miR-378c; and hsa-miR-4303, hsv2-miR-H13, and hsv2-miR-H10 were first reported with downregulation in gastric cancer. We provide new miRNA expression profiles of gastric cancer (Tables 1 and 2).

### 3.2. Quality Assessment of miRNA Data after Filtering

**Figure 1:**
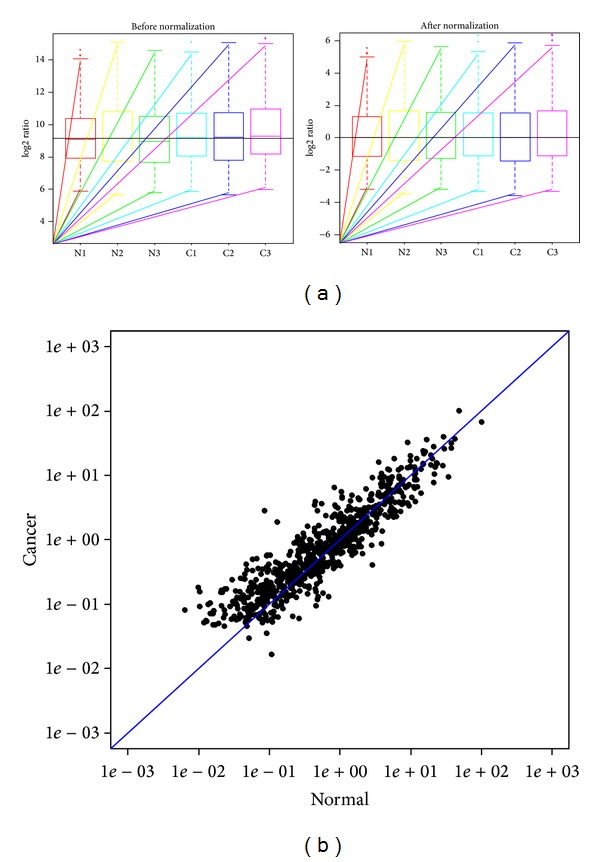
(a) The box plots are used to compare distributions of samples (left: nonnormalized log2-ratio data; right: median normalized log2-ratio data). (b) The scatter-plots assess the variation of miRNAs expression between cancerous and normal tissues. The axes of the scatter-plot are the normalized signal values of the samples (ratio scale).

To assess miRNA data, we built box plots to visualize the distribution of the miRNA dataset. Among six miRNA array chips, the distributions of log2 ratios are nearly the same (Figure 1(a)). Then, we apply correlation matrix to elevate correlation among replicate experiments. The scatter-plot was used to assess the variation between cancer and normal tissues. There are more than 80% of the same miRNA genes between cancerous and normal tissues (Figure 1(b)).

### 3.3. Clustering Analysis of the Significantly Changed Genes

**Figure 2:**
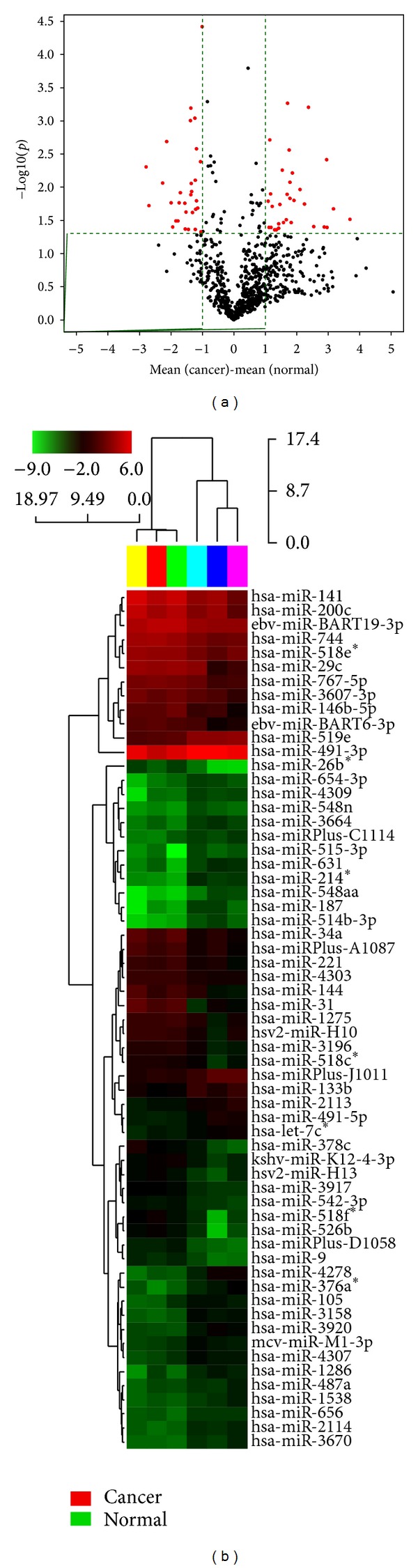
(a) Volcano plot showing the relative expression of miRNA genes form a one-class *t*-test. The vertical lines correspond to 2.0-fold up and down, respectively, and the horizontal line represents a *P* value of 0.05. The red point in the plot represents the differentially expressed genes with statistical significance. (b) Hierarchical clustering for differentially expressed miRNAs in cancer versus normal pass volcano plot (fold change ≥2.0). Red indicates high relative expression, and green indicates low relative expression.

To identify differentially expressed miRNAs with statistical significance, we performed a volcano plot filtering between the cancerous and normal miRNAs from the experiment (Figure 2(a)). The threshold we used to screen up- or downregulated miRNAs is fold change ≥2.0 and *P* value <0.05. As shown in Figure 2(a), the red points in the plot represent the differentially expressed genes with statistical significance. In this instance, we identified 182 commonly expressed miRNA genes. From this set, 31 were highly expressed and 30 showed low levels of expression across the cancerous and normal tissues. The following hierarchical clustering was performed based on differentially expressed miRNA in cancerous versus normal volcano plot. The result of hierarchical clustering shows distinguishable miRNA expression profiling among cancerous and normal tissues (Figure 2(b)). We set the *P* value at <0.05 as a cut-off level. Expression levels of 31 upregulated genes and 30 downregulated genes were analyzed by unsupervised hierarchical clustering. Our data show that all 61 miRNAs express similar patterns. The heat-map demonstrated that all these genes changed similarly in the different pairs of gastric cancerous and normal tissues (Figure 2(b)). The aberrantly expression miRNAs such as has-miR-214*, has-miR-105, has-miR-548n, and has-miR-514 which are upregulate in gastric cancerous tissues.

### 3.4. Gene Ontology Analysis

**Figure 3:**
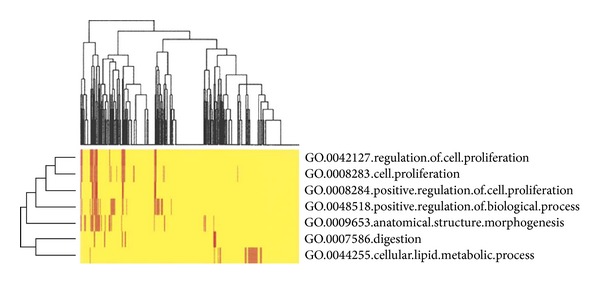
Clustering of overrepresented gene ontology (GO) classes in predicted targets of differential miRNAs.

The differentially expressed miRNAs would be expected to be significant to gastric cancer biology. Gene ontology (GO) analysis was applied to examine the significant “biological process” classifications that are overrepresented among these genes. This analysis revealed that many of the genes associated with tumor cell proliferation mainly take part in the processes of regulation of cell proliferation and maintain cell morphogenesis, digestion, and metabolism (Figure 3).

### 3.5. Validation of Aberrantly Expressed miRNA by Quantitative PCR Analysis

**Figure 4:**
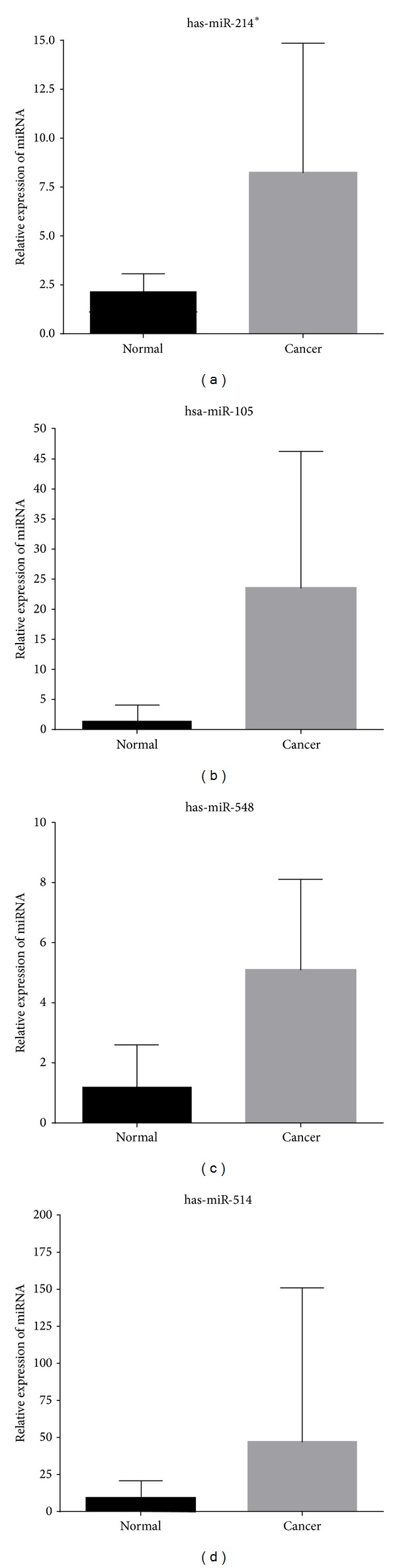
The miRNA expression level of has-miR-214* (a), has-miR-105 (b), has-miR-548n (c), and has-miR-514b (d) detected by qRT-PCR in cancerous and normal tissues. Relative gene expression was calculated as 2^−ΔΔCT^.

To validate the findings from expression arrays, four miRNAs were tested by real-time quantitative PCR analysis. We selected hsa-miR-105, hsa-miR-214*, hsa-miR-514b, and has-miR-548n to test in 24 paired gastric normal and tumor tissues. Control miRNA was U6. For every miRNA that was upregulated expression by microarray. The expression level of miR-214* is increased 3.79-fold (Figure 4(a)), miR-105 is increased 16.32-fold (Figure 4(b)), has-miR-548 is 4.21-fold (Figure 4(c)), and miR-514 is increased 11.76-fold (Figure 4(d)) in comparing with normal tissues. Those are inconsistent with the result of miRNA chip.

## 4. Discussion

Gastric cancer should be viewed as a heterogeneous disease, showing multiple biological and clinical differences. Recent several studies have shown the dysregulation of some miRNA in gastric cancer [16–18]. Previous high-throughput array analyses have clearly demonstrated that the miRNA expression profile is substantially altered in cancer samples [19]. Herein, we report comprehensive miRNA profiling in gastric cancer. In this study, we identified 61 miRNAs that are aberrantly expressed in gastric cancer. Comparing with other studies, we applied the microarray with the most huge detectable miRNA library with 1891 capture probes. As a result, a total of 31 miRNAs were upregulated in cancerous tissues, whereas 30 miRNAs were downregulated. Usually we set miRNA expression change at 2-fold as a cut-off, and this may neglect aberrant miRNAs in the chip analysis. Li et al. had established miRNA profiles of intestinal-type gastric cancer with 76 aberrantly expressed miRNAs [6]. The miRNA array chip of this study detected those 76 miRNA capture probes. However, only eight aberrantly expressed miRNAs (including has-miRplus-A1087, has-miR-542-3p, has-miR-141, has-miR-200c, has-miR-214, has-miR-29c, has-miR-378, and has-miR-128) were consistent with our findings. The reasons for discrepancy of two studies might be derived from different tumor types and heterogeneous. On the other hand, most of miRNA genes in our study might be newly detected in gastric cancer tissue and refer to important pathways during progress of carcinogenesis.

Some of the differentially expressed miRNAs in gastric cancer were proved to be aberrantly by other studies.* Helicobacter pylori* (*H. pylori*) infection is one of the most prevalent infections worldwide and has been identified as the major cause of this malignant disease. A subset of miRNA reported to associate with* H. pylori* infection, such as has-miR-141, has-miR-146a, and has-miR-2114, is detected in our study. The has-miR-2114 was reported to be upregulated, while has-miR-141 and has-miR-146a were downregulated [20]. This finding coincided with our results (Tables 1 and 2). Guo J et al. identified 19 differential miRNAs in gastric cancer tissues. Among them hsa-miR-31 and has-miR-133b were included which were screened in our findings [21]. In the present study, the miRNA chip revealed that the has-miR-31 was downregulated in gastric cancer tissues, consistent with a previous study by Zhang et al., which had identified that has-miR-31 was lower in cancer tissues in comparison with noncancerous tissues.

The metastases and invasions are the most important characteristics of malignant tumor. A lot of miRNAs genes detected in our study have been found to refer to those progresses by other studies and might serve as biomarkers to reflect disease state. has-miR-31 was frequently altered in a large variety of cancers. For example, in breast cancer loss of has-miR-31 expression is associated with high risk metastases [22], whereas in colorectal cancer high has-miR-31 expression correlates with advanced disease stage [23]. In the present study, has-miR-200c and has-miR-221 were downregulated and consistent with previous research [24], which confirmed that the downregulation of has-miR-200c and has-miR-221 could serve as one diagnostic biomarker of gastric cancer [25]. The miR-187 was increased in breast cancer tissues which could be used as one of the independent prognostic factors and enforced tumor cells invasive ability [26]. The accumulation of unedited has-miR-376a* was associated with glioma tumor metastasis and promoted cell migration and invasions [27]. Wang et al. proved that has-let-7c* inhibited migration and invasion of non-small cell lung cancer by targeting ITGB3 and MAP4K3 [28]. The p21 as tumor inhibitor had been explored; the overexpression of has-miR-515-3p rescued human mammary epithelial cells from Ras-induced senescence by prevention of Ras-induced upregulation of p21 [29]. The alterations in miRNA expression contributed to response chemotherapy. The has-miR-487a could directly regulate breast cancer resistance protein (BCRP) expression and reverse chemotherapeutic drug resistance in a subset of breast cancers [30]. has-miR-546b-5p had inhibitory effect on pancreatic cancer cell migration and invasion by targeting MMP16 [31].

Expression levels of four miRNAs were analyzed by quantitative real-time PCR. The results are consistent with miRNAs chip. All of them were upregulated in gastric cancerous tissues. The has-miR-105 was highly expressed in testis tumors [32]. The inhibitor efforts of has-miR-214 had been reported in several types of tumors, including hepatocellular carcinoma, cholangiocarcinoma, and ovarian cancer [33–35]. has-miR-214 had multiple roles in regulating tumor cell characteristics, such as proliferation and migration by targeting p53 and *β*-catenin [36]. Only one report by Wotschofsky et al. detected has-miR-514b and identified that the expression of has-miR-514b was downregulated in primary metastatic renal cell carcinoma [37]. At last, the expression level of has-miR-548n was upregulated in gastric cancer. The aberrantly of has-miR-548n may be associated with host antiviral response via direct targeting of interferon-*γ* [38].

By targeting tens to hands of genes, miRNAs can redirect basic biological functions and pathway essential to tumor development and progression. According to GO analysis, the major roles of aberrantly expressed miRNA regulated cell proliferation (Figure 3). The aberrantly expressed miRNAs of gastric cancer had been associated with regulating cell. We detected the downregulation of has-miR-9 in gastric cancer, which suppressed the proliferation, invasion, and metastasis of gastric cancer cells through targeting cyclin D1 and Ets1 [39]. Another miRNA gene, miR-144, was downregulated and had reported increased bladder cancer cell proliferation by targeting EZH2 and regulating Wnt signaling [40]. Furthermore, miRNAs served as mediators of inflammation in tumor progression through regulation of components of immune system. For example, the high levels of has-miR-187 promoted lymph node metastasis of breast cancer [26].

We also found some new aberrant expression miRNAs in gastric cancers (Tables 1 and 2), which have no or very few reports of aberrant expression in any other cancers. These new aberrant miRNAs are dysregulated in gastric cancer, ant the impact on gene expression. As more miRNAs are identified and validated, the role of aberrant miRNA expression in gastric cancer will be better understood. Our data may provide diagnostic or prognostic biomarkers of gastric cancer and offer new molecular targets for therapy of gastric cancer.

## Figures and Tables

**Table 1:** List of upregulated expression miRNAs in gastric cancer.

Gene name	Fold change	*P* value
hsa-miR-105	3.4367019	0.0084407
hsa-miR-4309	3.6494686	0.0061254
hsa-miR-3664	3.2239638	0.0127328
hsa-miR-187	7.3210044	0.0399018
hsa-miR-4307	2.9131846	0.0054889
hsa-miR-519e	3.2802326	0.0005427
hsa-miR-631	4.7584436	0.0180121
hsa-miR-491-5p	2.6810687	0.0411691
hsa-miR-4278	7.7996863	0.0400128
hsa-miR-548n	2.9842763	0.0338838
hsa-miR-514b-3p	9.0201538	0.0210931
hsa-miR-3920	4.2992771	0.0108692
hsa-miR-376a*	5.8401803	0.0390177
hsa-miR-214*	7.7435982	0.0038199
hsa-miRPlus-J1011	2.5587661	0.0436641
hsa-miR-3158	5.2006402	0.0006224
hsa-miR-1286	3.7748058	0.0156883
hsa-let-7c*	2.2461093	0.0126919
hsa-miR-654-3p	3.1872897	0.0308998
hsa-miR-1538	2.6964967	0.0355049
hsa-miR-515-3p	3.5260133	0.0339529
hsa-miR-2114	3.390285	0.0027316
hsa-miR-487a	2.1721574	0.0405062
mcv-miR-M1-3p	2.320875	0.0195074
hsa-miR-2113	2.4646054	0.0439924
hsa-miRPlus-C1114	2.7597195	0.0181086
hsa-miR-133b	2.2714611	0.0404879
hsa-miR-3670	3.4452207	0.0147389
hsa-miR-491-3p	2.1276933	0.0162673
hsa-miR-548aa	12.978879	0.0303442
hsa-miR-656	2.2199092	0.0019275

**Table 2:** List of downregulated expression miRNA in gastric cancer.

Gene name	Fold change	*P* value
hsa-miR-31	0.1444692	0.004953
hsa-miR-1275	0.3939808	0.011642
hsa-miR-26b*	0.1534911	0.0189828
hsa-miR-744	0.3825669	0.0009822
hsa-miR-146b-5p	0.3483215	0.0237032
hsa-miR-767-5p	0.4524493	0.0206438
hsv2-miR-H13	0.3396123	0.017379
hsa-miR-526b	0.2088823	0.0086282
ebv-miR-BART19-3p	0.4779629	0.0041381
hsa-miR-518f*	0.2507089	0.0171976
hsa-miR-3196	0.3889389	0.0128937
hsa-miR-3607-3p	0.4830454	0.0471262
hsa-miR-542-3p	0.4221895	0.0009132
hsa-miRPlus-A1087	0.4015859	0.0240223
hsa-miR-518c*	0.4395746	0.0160217
hsv2-miR-H10	0.3909028	0.0087108
hsa-miR-221	0.4285778	0.0077903
kshv-miR-K12-4-3p	0.4321184	0.0215663
hsa-miR-144	0.2929966	0.0320539
hsa-miR-9	0.3090372	0.0120556
hsa-miR-4303	0.4970407	3.813*E* − 05
hsa-miR-200c	0.3684039	0.0433953
hsa-miR-3917	0.3883737	0.000637
hsa-miR-29c	0.341262	0.0424996
ebv-miR-BART6-3p	0.4265503	0.0434011
hsa-miR-518e*	0.4387169	0.002632
hsa-miR-141	0.278258	0.032224
hsa-miR-34a	0.2989643	0.0172656
hsa-miRPlus-D1058	0.2284739	0.002042
hsa-miR-378c	0.2607431	0.0395669
